# Pedagogical foundations of cybercivility in health professions education: a scoping review

**DOI:** 10.1186/s12909-021-02507-z

**Published:** 2021-01-30

**Authors:** Jennie C. DE GAGNE, Paula D. KOPPEL, Sang Suk KIM, Hyeyoung K. PARK, Sharron RUSHTON

**Affiliations:** 1 School of Nursing, Duke University, Durham, NC USA; 2 Red Cross College of Nursing, Chung-Ang University, 84 Heukseok-ro, Dongjak-gu, Seoul, 06974 Republic of Korea; 3 College of Nursing, University of Massachusetts Amherst, Amherst, USA

**Keywords:** Cybercivility, Health professions education, Pedagogical foundation, Scoping review

## Abstract

**Background:**

Teaching cybercivility requires thoughtful attention to curriculum development and content delivery. Theories, models, and conceptual and theoretical frameworks (hereafter “tools”) provide useful foundations for integrating new knowledge and skills into existing professional practice and education. We conducted this scoping review to identify tools used for teaching cybercivility in health professions education.

**Methods:**

Using Arksey and O’Malley’s scoping review framework, we searched six biomedical and educational databases and three grey literature databases for articles available in English published between January 1, 2000 and March 31, 2020. Following the PRISMA-ScR (Preferred Reporting Items for Systematic Reviews and Meta-Analyses extension for Scoping Reviews), we screened and extracted relevant data, and reported the results of the search.

**Results:**

The search resulted in 2272 articles, with 8 articles included in this review after inclusion criteria were applied. Four articles (50%) were peer-reviewed journal papers while the other 4 (50%) were dissertations. Eleven unique tools were identified by this review: (1) Transpersonal Caring Theory, (2) Theory of Workplace Incivility, (3) Conceptualization of Incivility, (4) Media Ecology Theory, (5) Principlism, (6) Salmon’s Five Stage Model of Online Learning, (7) Learner-Centered Educational Theory, (8) Gallant and Drinan’s 4-Stage Model of Institutionalization of Academic Integrity, (9) Theory of Planned Behavior, (10) Communication Privacy Management Theory, and (11) Moral Development Theory. Based on the tools analyzed in our scoping review, we determined three features of cybercivility pedagogy to which the tools provided a guide: (1) behavioral manifestations, (2) academic integrity, and (3) digital professionalism.

**Conclusions:**

The reviewed tools provide a pedagogical foundation and guidance for teaching various properties of cybercivility. Future studies should be expanded to include a broader literature body and non-English literature to provide the global perspective and global skills needed by a diverse population of learners.

**Supplementary Information:**

The online version contains supplementary material available at 10.1186/s12909-021-02507-z.

## Background

Cybercivility is defined as respectful online interaction during computer-mediated communication. Cyberincivility refers to disrespectful, insensitive, or disruptive online misbehavior; the implications of cyberincivility for students’ psychological, physical, and social well-being in health professions education (HPE) as well as for their academic outcomes have been documented [1]. Cyberincivility in HPE includes but is not limited to academic dishonesty (e.g., plagiarism, lying, and cheating) [2–4] and disrespectful, insensitive, unhelpful, and unprofessional posts in online classroom discussion boards and on social media [1, 5, 6]. Recent studies show that most health professions students experience cyberincivility and see it as a moderate to serious problem [1, 6–8]. Given the increasing prevalence of online learning platforms, social networking, and digital communication, the need has never been higher for educators to adopt effective strategies to create civil and safe online learning environments. In order to create a safe place for all students, cybercivility should be taught and thoughtful attention should be given to curriculum development, content delivery, and administrative support related to policies and procedures [1, 9, 10].

Despite consistent research suggesting that cyberincivility can interfere with student learning in HPE [8, 9, 11], insufficient research has focused on the interventions on which development of a successful cybercivility curriculum or education plan should be based. In an effort to better understand specific areas of need or knowledge gaps pertaining to students’ cybercivility learning, an assessment of the needs was conducted with 205 students in medicine, nursing, physician assistant, and physical therapy programs [8]. The study reported that HPE students would benefit from lessons about cybercivility, and especially from learning about ethics and values that pertain to working in interprofessional teams [8]. The findings were consistent with those of a qualitative needs assessment with 25 HPE students [9]. Participants endorsed a variety of learning formats, including those within existing courses for core concepts and practical skills on cyberincivility [8]. Despite research that informs the necessity and importance of establishing cybercivility learning in HPE, the pedagogical foundations that could advance cybercivility education are not well known.

While theories, models, and conceptual and theoretical frameworks (hereafter “tools”) contain individual differences and similarities, the tools serve the same purpose in providing useful foundations when new knowledge and skills need to be integrated into existing professional practice and education [12–17]. Theoretical frameworks map knowledge about a phenomenon being studied [15], while conceptual frameworks illustrate the logic, relationships, and structure between ideas and concepts, and sets of values or beliefs [18, 19]. They provide a means for developing the depth and breadth of competencies and behaviors essential to health professions students [13]. Frameworks in education support conceptual consistency, theoretical foundations, unity, and continuity within the profession [14, 15]. They afford educators direction for building a curriculum by demonstrating the knowledge or skills that students need to attain [14, 15]. It is important for educators to assess, use, improve, and test the frameworks they select [14, 15]. Moreover, frameworks and theories are necessary for understanding the mechanisms of both behaviors and transformational interventions [15, 20]; therefore, we chose a scoping review methodology to identify the literature by searching for these strategic tools in the field being explored.

### Aim, objectives, and research questions

The aim of this scoping review was to examine conceptual and theoretical models that are relevant to the pedagogical aspects of teaching cybercivility to students of health professions. Specifically, we examined theories, models, and conceptual and theoretical frameworks used to understand the pedagogy of cybercivility in HPE. The research question of this study was: “What tools are relevant to the pedagogical aspects of cybercivility in HPE?” This review will (a) inform educators about applicable pedagogical foundations for cybercivility instruction in HPE, (b) benefit researchers in identifying knowledge gaps to construct new theories or models, and (c) aid educational practitioners in designing educational interventions and evaluation methods.

## Methods

Following the Joanna Briggs Institute (JBI) reviewers’ manual for scoping studies [21], we used the PCC (Population, Concept, and Context) mnemonic to establish an a priori protocol for defining the key terms, variables, and assumptions for this review [22]. For example, we considered studies related to health professions students exposed to cyberincivility, and we examined tools guiding research about health professions students and related to cyberincivility. The context of our review included academic, hospital, community, skills lab, and virtual classroom settings, or any other settings in which cyberincivility could take place, and virtual communications, including emails and blogs, online courses, and discussion forums. The framework by Arksey and O’Malley [23] guided the search strategy, data extraction process, and data reporting. Search terms used were based on an integrative review on cybercivility in health professions education [1] and included terms used in other disciples to describe cyberincivility such as cyberbullying and cyberharassment [24–26]. A protocol for the review was published in the JBI Database of Systematic Reviews and Implementation Reports [22, 23].

### Search strategy and study selection

We included peer-reviewed articles and grey literature available in English and published between January 1, 2000 and March 31, 2020. Conference abstracts and posters were excluded due to their brevity; editorials, commentaries, and opinion papers were not a consideration due to potential bias. For peer-reviewed articles, PubMed (MEDLINE), CINAHL (via EBSCO), Education Resources Information Center (ERIC via EBSCO), Embase (Elsevier), PsycINFO (via EBSCO), and Education Full Text (H.W. Wilson) databases were searched. For grey literature, we searched the ProQuest Dissertations and Theses Global database, OpenGrey, and EThOS. Additional file 1: Appendix I provides a list of search queries used in these databases. All identified citations from the search were uploaded into Covidence online software (Covidence, Melbourne, Victoria, Australia) for screening, and duplicates were removed by Covidence. Two researchers provided independent evaluations against the inclusion and exclusion criteria at the title and abstract level as well as at the full text level. Any disagreements that arose between the researchers during title, abstract, or full text screening were resolved through discussion or with a third reviewer. A critical appraisal of the evidence was not performed because it is generally not applicable to scoping reviews [27]. A PRISMA-ScR (Preferred Reporting Items for Systematic Reviews and Meta-Analyses extension for Scoping Reviews) checklist was used to report results of the search [27].

### Data extraction and synthesis

Studies included after the full text review underwent a process of data extraction, and each included item was abstracted by one researcher and reviewed by a second reviewer for accuracy and completeness. Any disagreements that arose between the researchers were resolved through discussion or with a third researcher during the extraction process. Guided by the JBI extraction instrument tool, which included study details, characteristics, and results [21], we prepared and filled in a data extraction matrix to chart and collate the final selected studies [22]. Texts pertinent to each heading in the matrix (e.g., author[s]/study year, country of origin, discipline, sample size, domain of cyber environment, participant characteristics, purpose of tool, etc.) were recorded, and the entries in each field were analyzed and synthesized to identify recurrent concepts. All selected studies for review researched health professions students and cybercivility; however, only two studies used the term “cyberincivility” as a whole as the key concept of research. To facilitate the synthesis of the literature on the tools used in various properties of cybercivility, we identified codes that emerged repeatedly in the reviewed studies and abstracted them into the overarching features of cybercivility pedagogy. Our process included both manual extraction and analysis using the matrix found in Additional file 1: Appendix II as well as computerized analysis of the extracted data using NVivo 12 (QSR International Pty Ltd., Doncaster, Victoria, Australia) software. To guide the computerized analysis, eight codes that emerged from the matrix data during the manual analysis were selected. These codes included caring, respect, behavior, integrity, change management, ethics, media communication, and professionalism. Each code was defined by the first coder (PK), and the second (HP) and third (JCD) coders reviewed the definitions of the eight codes and used them to search the text of each study included in the review systemically. Discrepancies in the coding were discussed during the research team meeting until agreement was met. A group of repeating or relating ideas in the codes were organized by relevance to three major categories: behavioral manifestations, academic integrity, and digital professionalism. This additional coding step was included in our analysis to ensure that all data relevant to the research question had been captured during the extraction process. We took a narrative approach to reporting the review findings, informed by our main research question.

## Results

**Fig. 1:**
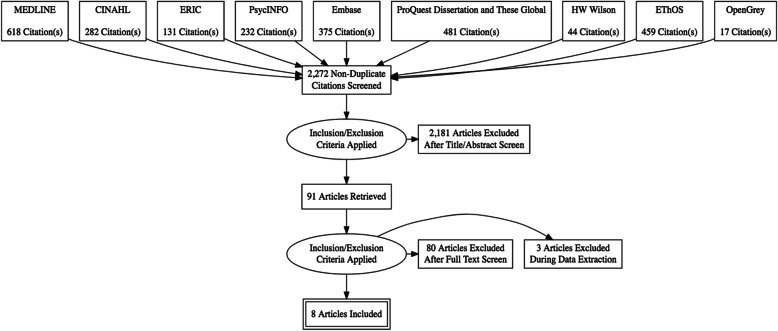
PRISMA-ScR (Preferred Reporting Items for Systematic Reviews and Meta-Analyses extension for Scoping Reviews) flow chart for the article search

Our search yielded 2272 potentially relevant studies from 9 databases after removing duplicates. Of the potentially relevant articles, 91 underwent a full-text review, and 83 (83/91, 91.2%) were excluded for the following reasons: absence of tool (38/83, 45.8%); inapplicable target population/audience (26/83, 31.3%); nonempirical literature such as editorial, commentary, or proceedings (14/83, 16.9%); inapplicable study setting (4/83, 4.8%); or duplicate article (1/83, 1.2%). Ultimately, 8 articles met the inclusion criteria (Fig. 1).

### Study characteristics

The selected articles (*n* = 8) were published between 2007 and 2018 [9, 28–34], and 7 of them (87.5%) were published after 2010 [9, 28, 30–34]. All studies (*n* = 8) were conducted in the United States [9, 28–34]. Four of the studies (50%) used a quantitative design [9, 28, 29, 34], and the other 4 studies (50%) used a qualitative approach [30–33]. Four articles (50%) were peer-reviewed journal papers [9, 29, 32, 33], and the other 4 (50%) were dissertations [28, 30, 31, 34] that required the use of a tool. The 8 studies reviewed included 1263 participants, mainly in the nursing discipline (*n* = 1252, 99%) [9, 28–34], and most of the nursing students were undergraduates (*n* = 1239, 98%) [28–34]. Although most participants were nursing students, other health-related disciplines were represented in the studies [9], including six (75%) that included medical students, three (38%) that included physical assistant students, and two (25%) that included students in physical therapy. The represented domains of cyber environment in the studies were online classrooms (*n* = 6, 75.0%) [28–33]; social networking sites such as Facebook, Twitter, Instagram, or Snapchat (*n* = 1, 12.5%) [34]; and general online settings including email, social networking sites, and online discussion forums (n = 1, 12.5%) [9].

The eight included studies assessed a variety of aims. Faculty behaviors were an identified outcome [28, 29] as well as factors underlying faculty behaviors [30]. Relationships were evaluated including a possible link between age, gender, and uncivil behaviors [30] as well as potential connection between academic and professional dishonesty [31]. Two studies looked at perceptions of incivility [9, 29]. Integrity was explored using survey methods [32, 33]. Finally, the process for maintaining e-professionalism was studied [34]. See Additional file 1: Appendix II for additional details.

### Tools identified in the literature of teaching cybercivility

Of the eight studies meeting our criteria, four (50.0%) referenced the use of models to facilitate an understanding of online behaviors and communication by students or faculty in HPE [9, 28–30]. Watson’s theory of transpersonal caring [35] was used to guide Bork’s study [28] exploring how male nursing students perceive caring and uncaring behaviors among online nursing faculty. Three of the 10 Caritas factors were highlighted: (a) developing self-awareness, (b) building relationship-based care and support, and (c) sharing teaching and learning experiences. These Caritas factors were seen as a potential guide for the development of words and behaviors which faculty could use to create a caring online environment between students and faculty [28]. On the other hand, Cain [30] combined two theories, the theory of workplace incivility [36] and the conceptualization of incivility [37], in order to examine the underlying factor structure and potential relationships between items in the Incivility Online Learning Environment (IOLE) survey, which measures student and faculty perceptions of incivility in the online learning environment. These theories provided a tool for enhanced understanding of how students and faculty perceive uncivil behaviors in an online learning environment and of how these behaviors impact teaching [30]. To better understand experiences of cyberincivility, De Gagne and colleagues [9] applied the media ecology theory [38] and the four principles of principlism [39]. Both tools have a strong ethical basis, and the media ecology theory offers a “humanistic perspective” on how online communication affects human perceptions, understandings, feelings, and values [9]. The principles of principlism, including autonomy, beneficence, nonmaleficence, and justice [39], are often included in health care professional curriculum and were viewed as potential guides for student behaviors. The two models, taken together, provided context for the study’s exploration of student experiences and learning needs relative to cyberincivility. The authors noted that these models could also guide development of educational resources [9]. Similarly, Rieck and Crouch [29] leveraged a 5-stage model by Salmon [40] and learner-centered educational theory [41] to guide an exploration of students’ perspectives of connectiveness among students as well as between the instructor’s behavior and civility in online nursing courses. While Salmon’s model [40] focuses on access and technology competence, interpersonal online skills, knowledge construction, and courteous interactions and communication, the learner-centered educational theory focuses more exclusively on interpersonal competency based on social interactions and respect for diversity [41].

Of the 8 studies included in our review, two (25.0%) utilized the 4-stage model for institutionalization of academic integrity [42] to guide their identification and implementation of an academic integrity intervention [32, 33]. Gallant and Drinan [42] outlined four stages: (a) recognition, (b) response generation, (c) response implementation, and (d) institutionalization of implementing academic integrity across an organization. Hart and Morgan [32] highlighted a faculty’s commitment to explore potential differences (stage 1 and 2) in the academic integrity of their asynchronous online and traditional nursing RN-BSN program cohorts [32]. Response implementation (stage 3) was illustrated on the outcomes of an academic integrity intervention [33]. This model supported the change process that faculty used to address the development and implementation of an academic integrity program for their online course. Similarly, one (12.5%) study focused on the development of an instrument for evaluating academic and professional dishonesty [31] using the theory of planned behavior [43]. This theory posits that an individual’s behaviors are shaped by intention toward attitude, norms, and perceived behavioral control.

Finally, one study (12.5%) employed a model to explore behaviors of information exchange on social media [34]: In Skrabal’s study [34], the communication privacy management (CPM) theory [44] and Kohlberg’s moral development theory [45] served as a foundation for understanding how undergraduate nursing students individually and collectively use social media. Skrabal’s study explored boundary formation, the generation of rules to govern boundaries, and the impact of “blurred” or “turbulent” boundaries on how nursing students manage their personal information to preserve e-professionalism [34]. The development of the Skrabal’s theory of e-professionalism [34] among prelicensure baccalaureate nursing students was attained through use of these theories and the author’s grounded theory research. Table 1 summarizes the purpose and details of each tool, the contribution to the body of knowledge in cybercivility, and its value in cybercivility pedagogy.

**Table 1:** Summary of tools identified in the articles reviewed (*n* = 8)

Author(s) (Year)	Purpose	Details	Contribution to the body of knowledge in cybercivility	Value in cybercivility curriculum
Bork (2014) [28]	To explore male nursing students’ perceptions of caring and uncaring behaviors by online nursing faculty, and of their interpretation and evaluation of behaviors identified in the study.	Watson’s transpersonal caring theory [35] has been used to describe a caring student-teacher relationship that enables the student to find meaning and wholeness to promote growth in nursing education. The 10 Caritas (carative factors): 1. Form a humanistic value system 2. Provide faith and hope 3. Develop self-awareness 4. Build relationships based on care and support 5. Promote and accept expression of feelings 6. Use the art of healing and caring with decision making and problem solving 7. Share genuine experiences of teaching and learning 8. Provide support and protection in multiple domains 9. Support basic human needs 10. Broaden an understanding of existential and phenomenological dimensions of self	Carative factor 3 (developing self-awareness about self and others) can help faculty to understand how to demonstrate caring in the online environment. Carative factor 4 (building relationship-based care and support) can guide faculty in building a supportive student relationship through facilitating an understanding of students’ unique needs. Carative factor 7 (sharing teaching and learning experiences) can inform educators of how gender impacts students’ perception of caring behaviors.	This tool can be used to provide guidelines around words, actions, and behaviors that are caring versus uncaring in an online environment. This research specifically focused on the student-teacher relationship. There is a need to intentionally include caring strategies by faculty in an online learning environment.
Cain (2017) [30]	To provide structure for the Incivility Online Learning Environment (IOLE) survey tests underlying factor structure in the faculty behavior items of the IOLE.	Andersson and Pearson’s theory of workplace incivility [36] defines incivility as rude or discourteous behaviors that disregard others and violate a universal workplace norm for respect. Per Clark et al.’s conceptualization of incivility in nursing education [37], uncivil behaviors result in physiological or psychological distress and can result in threatening situations. Clark’s IOLE survey [5] lists specific behaviors (actions or speech) that interfere with teaching and learning.	Andersson and Pearson’s theory [36] posits that low-intensity deviant behaviors should not be undermined as they could be more damaging to individuals than a single act of high-intensity behavior. Clark’s incivility in nursing education [37] guided the design of an instrument measuring incivility in an online learning environment.	Understanding what online behaviors students perceive as uncivil is important when creating a culture of civility in an online learning environment. Clark’s (2009) theory was used to determine the survey and the identification of two factors of uncivil faculty behavior factors, which the author denotes can lead to policy and faculty education. These theories provide a tool for viewing uncivil behaviors in an online classroom and may help educators.
De Gagne et al. (2018) [9]	To provide guidance for understanding cybercivility and cyberincivility experiences.	Postman’s theory of media ecology [38]: ethics with framework for evaluation of media environment, founded on an inquiry of how online communication affects human perceptions, understanding, feelings, and values by looking into the structure, content, and impact on social media users and their behavior. Beauchamp & Childress’s Principlism [39]: four principles including autonomy, beneficence, nonmaleficence, and justice	The media ecology theory offers a “humanistic perspective” on online misbehavior, thus facilitating behavioral and social management in the context of cyberspace. The four principles of principlism (i.e., autonomy, beneficence, nonmaleficence, and justice) have been used to teach ethical issues across curricula in health professions education and can serve as a powerful action guide for addressing student behaviors that may contribute to incivility.	The media ecology theory can guide development of relevant questions and of educational practice innovations for this environment. The four principles of principlism can be used to teach ethical issues across curricula in health professions education and can serve as a powerful action guide for addressing student behaviors that may contribute to incivility.
Hart & Morgan (2010) [32]	To guide faculty in institutionalizing academic integrity	Gallant and Drinan’s 4-stage model [42] for institutionalization of academic integrity: stage 1 = recognition stage 2 = response generation stage 3 = response implementation stage 4 = institutionalization The study design represents Stage 2 of this model.	Authors indicated that faculty had a dialogue about perceived differences in online and traditional classrooms (stage 1) and made a commitment to explore academic integrity in the two programs (stage 2).	All nursing programs should clearly delineate expectations for academic integrity for students in an online learning environment. Procedures to increase awareness of academic integrity policies should be put in place.
Morgan & Hart (2013) [33]	To develop an intervention involving faculty-facilitated discussion about academic integrity during the first week of class via an online discussion forum	Gallant and Drinan’s 4-stage model [42] for institutionalization of academic integrity: stage 1 = recognition stage 2 = response generation stage 3 = response implementation stage 4 = institutionalization	Authors developed an intervention that entailed encouraging students’ thoughts about the importance of academic integrity among themselves and with faculty. Students were instructed to read the testing policy statement and were required to acknowledge their understanding of its information prior to examinations.	Authors recommend that academic integrity become part of a culture. Academic integrity includes plagiarism, appropriate and inappropriate student collaboration, fabrication of information, and examination security. Their tool supported this concept.
Rieck & Crouch (2007) [29]	To guide a study design to explore students’ perspectives on connectiveness and civility in online nursing to increase effectiveness in online learning	Salmon’s 5-stage model of online learning [40]: access and technology competence, development of online interpersonal skills, knowledge construction, student achievement of courteous interactions, and communication Learner centered educational theory [41] is based on interpersonal competence and suggests that knowledge is built through social interaction and respect for diversity.	Authors emphasized the importance of online socialization in discussions, chats, emails, and blogs, or wikis.	The two strategic tools are of value to educators when creating a set of online classroom expectations, not only for students but for faculty (i.e., guidelines for civil communication apply to both students and faculty). It is important to provide prompt and meaningful feedback and post guidelines for civil communication and to provide examples.
Skrabal (2017) [34]	To provide guidance on sharing information on social media and to determine whether ethical development influences the use of social media among nursing students	Petronio’s communication privacy management theory (CPM) [44] with 5 suppositions: •Individuals have control over own information •Boundaries are created between private and public information •Information is owned or co-owned •Rules are used in information disclosure •Dialectical (tensions) Kohlberg’s moral development theory [45] •Pre-conventional morality: response to rules and labels •Conventional morality: conforms to norms and rules of a group such as family •Post-conventional morality: accepted societies rules and norms	CPM has an impact on how social media is used by students on e-professionalism, including establishment of boundaries and the rules that govern boundaries. In line with Kohlberg’s theory, ethical reasoning is an important aspect of communication and ethical behavior as well as of e-professionalism.	Nursing students use rules to manage e-professionalism consistent with CPM and ethical reasoning. Author notes that while students like the rules, rule-based education does not work. CPM and ethical reasoning can guide concept-based education using current guidelines.
Smith (2010) [31]	To guide evaluation of school’s handling of academic dishonesty issues, for instrument development, and to establish the relationship between academic dishonesty and professional dishonesty.	Theory of planned behavior (Beliefs influence behavior) [43] Concepts: •Behavioral belief •Normative beliefs •Control beliefs •Actual behavioral control •Behavioral intention	The theory was used to develop an instrument which can be used to understand academic and professional dishonesty. Framework can also be used to develop and test interventions.	Provided the framework for the development of the PAPIS (Perceptions of Academic and Professional Integrity Survey) The author points out that dishonest behaviors seem to start during the student experience.

### Tools and features of cybercivility

**Fig. 2:**
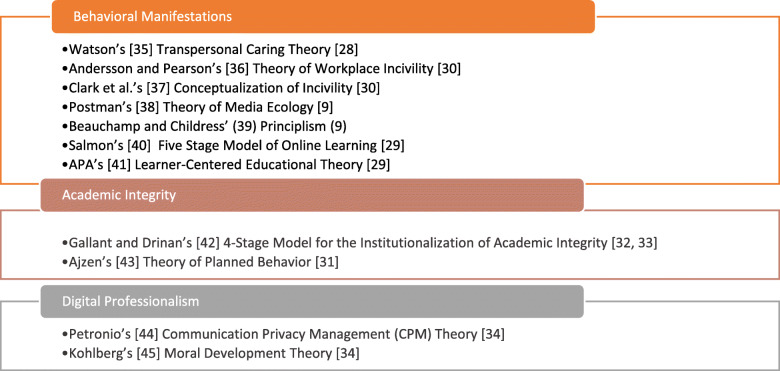
Identified tools by major feature of cybercivility

Each researcher identified three features of cybercivility pedagogy to which the tools provided a guide and then cross-referenced for validity with each extracted literature reference. While some of the tools incorporate more than one element (e.g. Kohlberg’s Moral Development Theory and Ajzen’s Theory of Planned Behavior both include behavioral manifestations) this figure reflects the tools that might be most useful in addressing each element. Figure 2 illustrates the relationship of the tools to three primary features important to teaching cybercivility: behavioral manifestations, academic integrity, and digital professionalism.

### Behavioral manifestations

Four of the eight studies (50%) focused on caring/uncaring or on civil/uncivil behaviors [9, 28–30]. These behaviors occurred within student-student interactions [9, 29] and student-teacher interactions [28–30], thus requiring a focus on both categories of relationships, their interconnections within the online platform, and the antecedents, attributes and outcomes of the behaviors. The studies we reviewed identified some of these qualities. For example, Bork [28] found that male nursing students perceived terse emails or unclear assignment instructions as indicators of an uncaring culture; this perception decreased their motivation to learn and increased the likelihood they would drop out or withdraw from the course. Moreover, Rieck and Crouch [29] found that students reported uncivil behavior among faculty 60% of the time, including behaviors that made students feel that their work was being belittled or that faculty were bothered when asked to respond to questions. Other characteristics that were identified in this review included potential explanations or antecedents for uncivil behaviors including anonymity, stress, lack of cues (verbal or nonverbal), workload, topics driven by passion, misunderstandings, and limited accountability, as well as cultural differences [9].

### Academic integrity

Sixty-eight percent (68%) of online RN to BSN students in one study admitted to having engaged in academic dishonesty, with the most common type of dishonesty cited being failure to contribute to group work [31]. In another set of studies, both online and traditional (on-campus classroom) students indicated that they infrequently cheated [32, 33]. Self-reported academic dishonesty was not correlated with age, gender, ethnicity, or work status in RN to BSN students [31]. Integrity violations may have been limited by the presence of an honor code and integrity policies [31, 33].

### Digital professionalism

Professionalism in computer-mediated communication was also explored within the included studies. Nursing students identified several types of online behaviors as being professional, such as creating positive posts, using appropriate language, and wearing appropriate attire for an online social media presence [34]. Additionally, students expressed an ability to maintain digital professionalism or e-professionalism by using privacy settings, utilizing separate accounts for personal and professional purposes, and not posting inappropriate information [31]. Skrabal [34] noted that strict rule-based education has its limitations, and other methods are important to consider, especially with adult learners. De Gagne et al. [1] suggested that fostering professional development requires not only cultivating knowledge and skill, but also socializing students within ethical and moral tools. Literature demonstrates that health professions students lack this knowledge and benefit from online courses, resources, and formats that facilitate discussions and reflections [1].

## Discussion

In our scoping review, the literature on cybercivility in health professions education was systematically searched and summarized to identify relevant tools and their implications on the development of cybercivility pedagogy. As addressed in the results section, we identified the following three features pertinent to cybercivility pedagogy: (1) behavioral manifestations, (2) academic integrity, and (3) digital professionalism.

### Behavioral manifestations

Our findings are consistent with those of studies in which respect or respectful and clear communication were identified by students as important qualities in their teachers as well as attributes that encouraged their learning [46, 47]. This would suggest that faculty should carefully consider their communication styles with a focus on delivering a clear message. Another pedagogical consideration is the capacity of teachers to be present during interactions with their students [48]. Further pedagogical considerations could be supported by a model consolidating research regarding antecedents to uncivil behaviors in online classrooms. Similarly, the studies reviewed revealed mechanisms for addressing uncivil behaviors including creating a positive culture with clear guidance provided by policies, procedures, and ongoing training [9], and handling such matters privately [29]. Additionally, educators can also implement a set of agreed upon behaviors (e.g. Netiquette) to facilitate online discussion [49, 50]. A model demonstrating how these interventions support desired outcomes could inform further teaching strategies and innovation.

Research on facilitating social presence in online learning environments increasingly demonstrates that specific strategies can cultivate a sense of caring, belonging, and safety that leads to mutual respect and meaningful relationships [51, 52]. For example, actions such as taking time to get to know students individually, encouraging students to share their personal experiences, facilitating connections between students, and acknowledging student contributions help to create a climate of trust, respect, and caring, even in cyberspace, by demonstrating that a teacher values the individuality of each student [52]. Incorporating these types of strategies into a model of cybercivility could help faculty to facilitate desired behavioral manifestations.

### Academic integrity

Academic dishonesty is another form of uncivil behavior that has been studied. Morgan and Hart [33] found that students’ understanding and compliance with academic integrity standards were enhanced through open discussions with faculty about the importance of academic integrity and about specific topics like plagiarism, appropriate and inappropriate collaboration, fabrication of information, and examination security [33]. This type of open dialog is in alignment with adult learning theories that suggest that active learning engagement and reflection are essential and are especially effective for learning in the affective domain because they allow students to reflect on their values and beliefs concerning their actions [10, 53].

Exploring why students may be prone to breaching academic integrity is an important question, and although it is not directly addressed by the studies in this review, a number of the theoretical and conceptual models used in the studies (i.e., theory of planned behavior [43], Kohlberg’s moral development theory [45], theory of media ecology [38] and principlism [39]) could provide a foundation for further research. Since principlism and moral development theory are often incorporated in HPE curriculum [54, 55], faculty may be receptive to using them to plan strategies for cultivating academic integrity.

### Digital professionalism

Elements of the theory of planned behavior [43], Kohlberg’s moral development theory [45] and Petronio’s [44] CPM theory could be used to enhance a more comprehensive cybercivility model and/or professional guidelines. The capacity to preserve professionalism was found to be influenced by an individual’s ability to manage emotions and reason ethically as well as by having received education pertaining to social media [34]. A conceptual model of cybercivility that reflects current strategies and supports the development of new pedagogical interventions is needed.

Albert Bandura’s social learning theory is a pedagogy associated with online learning that focuses, in part, on role modeling [50]. Role modeling is a powerful strategy often used to teach communication and professionalism to prelicensure nursing students [56, 57] and to socialize medical students [58]. While an integrated review of undergraduate nursing education research found that studies were focused primarily on nurse role modeling in the clinical setting rather than in academic settings [56], mentors in both settings can demonstrate professional behavior in a cyber environment to students.

The cyber environment, in all its various platforms, provides students with the opportunity to observe (and teachers with the chance to showcase) professional behaviors and civil online communication techniques (i.e., emails, tweets, discussion board posts). Case studies and online discussions allow students to share personal experiences and to brainstorm ways of responding to emotionally challenging clinical situations [10, 51]. The need to define and address consequences of uncivil behaviors, as well as the need to provide supportive resources both for individuals who enact uncivil behaviors and for the victims of these behaviors, has been described as being important to successful practice [10].

### Implications for a cybercivility pedagogical foundation

While no study we reviewed provided comprehensive support for the pedagogical foundation necessary to facilitate cybercivility in health professions education, the eight studies, taken together, provide insight and direction for the development of such a model. Watson’s transpersonal caring theory [35] provides a strong foundation for addressing the relationship aspects of cybercivility between students as well as between teachers and students. This theory also provides guidance for cultivating an environment of civility based on caring attitudes and behaviors. In contrast, the focus of Andersson and Pearson’s [36] and Clark et al.’s [37] theories is incivility. The media ecology theory [38] and the four principles of principlism [39] offer insights on the impact of online communication on human responses [9]. Taken together, perhaps these theoretical models can provide a more complete tool for (a) understanding uncivil behaviors, (b) helping faculty learn ways to cultivate caring environments and relationships within online platforms, and (c) guiding the development of curriculum, policies, and procedures that support caring and civil online learning experiences. Likewise, elements of Kohlberg’s moral development theory [45], principlism [39], the theory of media ecology [38], and the theory of planned behavior [43] could be used to address academic integrity as part of a model of cybercivility pedagogy.

Several of the tools utilized in the articles reviewed are already incorporated into health care curriculums. For example, Watson’s transpersonal caring theory is taught in schools of nursing, and Kohlberg’s moral development theory is often included in required ethics courses offered in a variety of HPE disciplines [54, 55, 59]. Leveraging existing tools to teach about cybercivility could give students the opportunity to understand these theories more deeply and could demonstrate how they might be applied pragmatically in students’ professional lives. Finally, while several of the articles we reviewed noted theories and models that could direct the development of guidelines and policies for both students and faculty [37, 40, 41, 44], the need for a comprehensive model seems essential in order to create an integrated approach that informs not only guidelines and policies but also HPE curriculum development, teaching strategies, and role modeling.

### Limitations

There are limitations to this scoping review. First, a small number of studies were identified, half of which were classified as grey literature. Although including grey literature could introduce bias into the results, we did so in order to explore the current evidence base fully. A formal risk of bias was not completed due to the nature of the review and the studies included, and because PRISMA-ScR denotes that appraisal only needs to be done if applicable [27]. Second, the use of tools was inconsistent, and the majority were only used once. Gallant and Drinan’s [42] 4 stage model for institutionalization of academic integrity was used by the same authors in two articles on academic integrity. Additionally, half of the studies used more than one tool. The use of multiple tools is not necessarily surprising given the complexity of cybercivility; more than one existing theory may be needed to explain the concept. Finally, all the included studies were completed in the United States, thus only a single country’s perspective on studying cybercivility with supporting tools is provided. This gap suggests that future research should attempt to provide a better understanding of cybercivility from a global perspective.

## Conclusion

Although civil behavior in cyberspace is an important element of online education, it can be challenging to teach civil behaviors, content, and process given subjective assumptions of what constitutes cybercivility and cyberincivility. Theories, models, and frameworks can provide useful guidance when new knowledge and skills are needed. Given the accepted importance of cultivating cybercivility in increasingly online educational environments, we were surprised to find only a few studies that included these types of tools. We reviewed strategic tools that could serve as pedagogical foundations and could provide guidance for educational practitioners designing curriculum and evaluation methods pertaining to cybercivility instruction in HPE; these tools address various features of cybercivility such as academic integrity and digital professionalism. As civility in the classroom needs to be defined, taught, and fostered, the use of well-designed programs of instruction on cybercivility and cyberincivility is critical for promoting ethical knowledge and skills through formal and informal curricula.

## Supplementary Information

**Additional file 1: Appendix I: Search Strategy Report. Appendix II.** Summary of included studies and main findings (*n* = 8)

## Data Availability

The dataset supporting the conclusions of this article is included within this article and its additional files as appendixes.
