# Supplementary material for: Pedagogical foundations of cybercivility in health professions education: a scoping review
Source: BMC Med Educ. 2021 Jan 30;21:79. doi: 10.1186/s12909-021-02507-z (PMC7847571; doi:10.1186/s12909-021-02507-z)
Supplement: Supplementary file 1 — Additional file 1: Appendix I: Search Strategy Report. Appendix II. Summary of included studies and main findings (n = 8) [file 12909_2021_2507_MOESM1_ESM.docx]

**Appendix I: Search Strategy Report**

**Database: PubMed (MEDLINE)**

| Set # |  | Results |
| --- | --- | --- |
| 1 | Cyber[tiab] OR "Internet"[mesh] OR "Education, Distance"[Mesh] OR "distance"[tiab] OR "internet"[tiab] OR Online[tiab] OR Web[tiab] OR Forum[tiab] OR Forums[tiab] OR "Discussion Board"[tiab] OR "Discussion Boards"[tiab] | 447,068 |
| 2 | “Cyber-bullying”[tiab] OR “cyberbullying”[tiab] OR "cyber aggression"[tiab] OR “cyber-incivility”[tiab] OR "Aggression"[Mesh] OR Hostility[Mesh] OR "Anger"[Mesh] OR bullying[tiab] OR bully[tiab] OR bullies[tiab] OR bullied[tiab] OR hostility[tiab] OR hostile[tiab] OR aggression[tiab] OR aggressive[tiab] OR civility[tiab] OR civilized[tiab] OR civilised[tiab] OR incivility[tiab] OR uncivil[tiab] OR disrespect[tiab] OR disrespectful[tiab] OR Rude[tiab] OR Rudeness[tiab] OR Haze[tiab] OR Hazing[tiab] OR belittle[tiab] OR belittling[tiab] OR Demean[tiab] OR Demeaning[tiab] OR Harass[tiab] OR harassing[tiab] OR Anger[tiab] OR Vulgar[tiab] OR Vulgarity[tiab] OR Inappropriate[tiab] OR Trolling[tiab] OR Troll[tiab] OR unprofessional[tiab] OR professionalism[tiab] OR misconduct[tiab] OR misbehavior[tiab] OR misbehaviors[tiab] OR misbehave OR “interpersonal deviance”[tiab] OR disruptive[tiab] | 293,808 |
| 3 | "Education, Professional"[Mesh] OR "Schools, Health Occupations"[Mesh] OR "Students, Health Occupations"[mesh] OR "Faculty, Nursing"[Mesh] OR "Faculty, Medical"[mesh] OR "Faculty, Dental"[Mesh] OR ((Nursing[tiab] OR medical[tiab] OR dental[tiab] OR pharmacy[tiab] OR "public health"[tiab] OR "allied health"[tiab] OR midwifery[tiab]) AND (education[tiab] OR school[tiab] OR schools[tiab] OR student[tiab] OR students[tiab] OR faculty[tiab] OR universities[tiab] OR university[tiab] OR "Curriculum"[Mesh] OR curriculum[tiab] OR curricula[tiab] OR curricular[tiab] OR lesson[tiab] OR lessons[tiab] OR "instructional design"[tiab])) | 552,234 |
| 5 | #1 AND #2 AND #3 | 748 |
| 6 | #5 NOT (((teens[tiab] OR teen[tiab] OR teenager[tiab] OR adolescent[tiab] OR adolescents[tiab] OR adolescence[tiab] OR child[tiab] OR kid[tiab] OR kids[tiab] OR children[tiab] OR youth[tiab] OR “high school”[tiab] OR infant[tiab] OR newborn[tiab] OR adolescent[Mesh] OR child[Mesh] OR infant[Mesh])) NOT "Adult"[Mesh]) | 671 |
| 7 | #6 AND English[lang] | 659 |
| 8^a^ | #7 AND ( ( "2007/01/01"[PDat] : "2019/12/31"[PDat] ) ) | 584 |
| 9 | Additional articles added with update (to reach 584) | 16 |
| 10 | Additional articles added with update Jan 1, 2020 – March 31, 2020 | 34 |

^a^The year 2007 was used instead of 2000 because the first reference to cyberincivility was found at the beginning of 2007 in an integrative review of cybercivility in HEP [1].

**Database: CINAHL Complete**

| Set # |  | Results |
| --- | --- | --- |
| 1 | MH "Internet" OR MH "Education, Non-Traditional" OR TI (Cyber OR distance OR "internet" OR Online OR Web OR Forum OR Forums OR "Discussion Board" OR "Discussion Boards") OR AB (Cyber OR distance OR "internet" OR Online OR Web OR Forum OR Forums OR "Discussion Board" OR "Discussion Boards") | 165,610 |
| 2 | TI (Cyber-bullying OR cyberbullying OR "cyber aggression" OR “cyber-incivility” OR bullying OR bully OR bullies OR bullied OR hostility OR hostile OR aggression OR aggressive OR civility OR civilized OR civilised OR incivility OR uncivil OR disrespect OR disrespectful OR Rude OR Rudeness OR Haze OR Hazing OR belittle OR belittling OR Demean OR Demeaning OR Harass OR harassing OR Anger OR Vulgar OR Vulgarity OR Inappropriate OR Trolling OR Troll OR unprofessional OR professionalism OR misconduct OR misbehavior OR misbehaviors OR misbehave OR “interpersonal deviance” OR disruptive) OR AB (Cyber-bullying OR cyberbullying OR "cyber aggression" OR “cyber-incivility” OR bullying OR bully OR bullies OR bullied OR hostility OR hostile OR aggression OR aggressive OR civility OR civilized OR civilised OR incivility OR uncivil OR disrespect OR disrespectful OR Rude OR Rudeness OR Haze OR Hazing OR belittle OR belittling OR Demean OR Demeaning OR Harass OR harassing OR Anger OR Vulgar OR Vulgarity OR Inappropriate OR Trolling OR Troll OR unprofessional OR professionalism OR misconduct OR misbehavior OR misbehaviors OR misbehave OR “interpersonal deviance” OR disruptive) OR MH "aggression+" OR MH "anger" OR MH "Student Misconduct" | 123,388 |
| 3 | MH "Education, Health Sciences" OR "Schools, Health Occupations" OR MH "Students, Health Occupations" OR MH "Faculty, Health Occupations" OR TI ((Nursing OR medical OR "allied health" OR dental OR midwifery OR pharmacy OR "public health") AND (education OR school OR schools OR student OR students OR faculty OR universities OR university)) OR AB ((Nursing OR medical OR "allied health" OR dental OR midwifery OR pharmacy OR "public health") AND (education OR school OR schools OR student OR students OR faculty OR universities OR university)) | 150,909 |
| 4 | #1 AND #2 AND #3 | 368 |
| 5 | #4 NOT ((((TI (teens OR teen OR teenager OR adolescent OR adolescence OR adolescents OR child OR kid OR kids OR children OR youth OR “high school” OR infant OR newborn) OR AB (teens OR teen OR teenager OR adolescent OR adolescence OR adolescents OR child OR kid OR kids OR children OR youth OR “high school” OR infant OR newborn) OR MH "Adolescence" OR MH "Child")) NOT (MH "Adult+")) | 316 |
| 6 | #6 AND LA English | 309 |
| 7^a^ | #7 limited to publication date from 1/1/2007 to 12/31/2020 | 268 |
| 8 | Additional articles added with update Jan 1, 2020 – March 31, 2020 | 14 |

^a^The year 2007 was used instead of 2000 because the first reference to cyberincivility was found at the beginning of 2007 in a integrative review of cybercivility in HEP [1].

**Database: ERIC**

| Set # |  | Results |
| --- | --- | --- |
| 1 | DE "Internet" OR DE "Distance Education" OR DE "Nontraditional Education" OR DE "Online Courses" OR DE "Virtual Classrooms" OR DE "Virtual Universities" OR DE "Blended Learning" OR DE "Web Based Instruction" TI (Cyber OR distance OR "internet" OR Online OR Web OR Forum OR Forums OR "Discussion Board" OR "Discussion Boards") OR AB (Cyber OR distance OR "internet" OR Online OR Web OR Forum OR Forums OR "Discussion Board" OR "Discussion Boards") | 131,349 |
| 2 | TI (Cyber-bullying OR cyberbullying OR "cyber aggression" OR “cyber-incivility” OR bullying OR bully OR bullies OR bullied OR hostility OR hostile OR aggression OR aggressive OR civility OR civilized OR civilised OR incivility OR uncivil OR disrespect OR disrespectful OR Rude OR Rudeness OR Haze OR Hazing OR belittle OR belittling OR Demean OR Demeaning OR Harass OR harassing OR Anger OR Vulgar OR Vulgarity OR Inappropriate OR Trolling OR Troll OR unprofessional OR professionalism OR misconduct OR “interpersonal deviance” OR misbehavior OR misbehaviors OR misbehave OR disruptive) OR AB (Cyber-bullying OR cyberbullying OR "cyber aggression" OR “cyber-incivility” OR bullying OR bully OR bullies OR bullied OR hostility OR hostile OR aggression OR aggressive OR civility OR civilized OR civilised OR incivility OR uncivil OR disrespect OR disrespectful OR Rude OR Rudeness OR Haze OR Hazing OR belittle OR belittling OR Demean OR Demeaning OR Harass OR harassing OR Anger OR Vulgar OR Vulgarity OR Inappropriate OR Trolling OR Troll OR unprofessional OR professionalism OR misconduct OR “interpersonal deviance” OR misbehavior OR misbehaviors OR misbehave OR disruptive) OR DE "bullying" OR DE "aggression" OR DE "hazing" OR DE “Teacher Behavior” OR DE “Student Behavior” OR DE "Student Reaction" OR DE "Professionalism" | 63,690 |
| 3 | DE "Medical Education" OR DE "Graduate Medical Education" OR DE "Nursing Education" OR DE "Pharmaceutical Education" OR DE "Allied Health Occupations Education" OR DE "Medical Schools" OR DE "Dental Schools" OR DE "Universities" OR DE "Nursing Students" OR DE ”medical students” OR DE "Medical School Faculty" OR TI ((Nursing OR medical OR "allied health" OR dental OR midwifery OR pharmacy OR "public health") AND (education OR school OR schools OR student OR students OR faculty OR universities OR university)) OR AB ((Nursing OR medical OR "allied health" OR dental OR midwifery OR pharmacy OR "public health") AND (education OR school OR schools OR student OR students OR faculty OR universities OR university)) | 52,375 |
| 4 | #1 AND #2 AND #3 | 198 |
| 5 | #4 NOT ((TI (teens OR teen OR teenager OR adolescent OR adolescence OR adolescents OR child OR kid OR kids OR children OR youth OR “high school” OR infant OR newborn) OR AB (teens OR teen OR teenager OR adolescent OR adolescence OR adolescents OR child OR kid OR kids OR children OR youth OR “high school” OR infant OR newborn) OR DE "Adolescents" OR DE "Children" OR DE "Early Adolescents")) NOT DE "Adults") | 156 |
| 6 | #5 AND LA English | 153 |
| 7 | Limit publication date to 2007-2019 | 131 |
| 8 | Additional articles added with update Jan 1, 2020 – March 31, 2020 | 0 |

**Database: PsycINFO**

| Set # |  | Results |
| --- | --- | --- |
| 1 | DE "Internet" OR DE "Blog" OR DE "Distance Education" OR DE "Virtual Classrooms" OR TI (Cyber OR distance OR "internet" OR Online OR Web OR Forum OR Forums OR "Discussion Board" OR "Discussion Boards") OR AB (Cyber OR distance OR "internet" OR Online OR Web OR Forum OR Forums OR "Discussion Board" OR "Discussion Boards") | 167,538 |
| 2 | TI (Cyber-bullying OR cyberbullying OR "cyber aggression" OR “cyber-incivility” OR bullying OR bully OR bullies OR bullied OR hostility OR hostile OR aggression OR aggressive OR civility OR civilized OR civilised OR incivility OR uncivil OR disrespect OR disrespectful OR Rude OR Rudeness OR Haze OR Hazing OR belittle OR belittling OR Demean OR Demeaning OR Harass OR harassing OR Anger OR Vulgar OR Vulgarity OR Inappropriate OR Trolling OR Troll OR unprofessional OR professionalism OR misconduct OR “interpersonal deviance” " OR misbehavior OR misbehaviors OR misbehave OR disruptive) OR AB (Cyber-bullying OR cyberbullying OR "cyber aggression" OR “cyber-incivility” OR bullying OR bully OR bullies OR bullied OR hostility OR hostile OR aggression OR aggressive OR civility OR civilized OR civilised OR incivility OR uncivil OR disrespect OR disrespectful OR Rude OR Rudeness OR Haze OR Hazing OR belittle OR belittling OR Demean OR Demeaning OR Harass OR harassing OR Anger OR Vulgar OR Vulgarity OR Inappropriate OR Trolling OR Troll OR unprofessional OR professionalism OR misconduct OR “interpersonal deviance” OR misbehavior OR misbehaviors OR misbehave OR disruptive) OR DE "bullying" OR DE "aggression" OR DE "hazing" OR DE "Aggressive Behavior" OR DE "Attack Behavior" OR DE "Coercion" OR DE "Conflict" OR DE "Microaggression" OR DE "Relational Aggression" OR DE "Anger" OR DE "Hostility" | 164,434 |
| 3 | DE "Medical Education" OR DE "Medical Internship" OR DE "Medical Residency" OR DE "Nursing Education" OR DE "Graduate Schools" OR DE "Graduate Students" OR DE "Medical Students" OR DE "Nursing Students" OR DE "Dental Students" OR DE "Graduate Schools" OR DE "Higher Education" OR DE "Graduate Education" OR DE "Postgraduate Training" OR DE "Undergraduate Education" OR DE "College Teachers" OR TI ((Nursing OR medical OR "allied health" OR dental OR midwifery OR pharmacy OR "public health") AND (education OR school OR schools OR student OR students OR faculty OR universities OR university)) OR AB ((Nursing OR medical OR "allied health" OR dental OR midwifery OR pharmacy OR "public health") AND (education OR school OR schools OR student OR students OR faculty OR universities OR university)) | 117,061 |
| 4 | #1 AND #2 AND #3 | 322 |
| 5 | #4 NOT ((TI (teens OR teen OR teenager OR adolescent OR adolescence OR adolescents OR child OR kid OR kids OR children OR youth OR “high school” OR infant OR newborn) OR AB (teens OR teen OR teenager OR adolescent OR adolescence OR adolescents OR child OR kid OR kids OR children OR youth OR “high school” OR infant OR newborn)) NOT (TI(adult or adults) OR AB(adult or adults)) | 236 |
| 6 | #5 AND LA English | 233 |
| 7 | #6 limited to publication date 2000-2019 | 231 |
| 8 | Additional articles added with update Jan 1, 2020 – March 31, 2020 | 1 |

**Database: Embase**

| Set # |  | Results |
| --- | --- | --- |
| 1 | Cyber:ti,ab OR 'Internet'/exp OR 'distance education'/exp OR 'internet':ti,ab OR Online:ti,ab OR Web:ti,ab OR distance:ti,ab OR Forum:ti,ab OR Forums:ti,ab OR 'Discussion Board':ti,ab OR 'Discussion Boards':ti,ab | 552,725 |
| 2 | 'aggression'/exp OR 'cyberbullying'/exp OR 'Cyber-bullying':ti,ab OR 'cyberbullying':ti,ab OR 'cyber aggression':ti,ab OR 'cyber-incivility':ti,ab OR bullying:ti,ab OR bully:ti,ab OR bullies:ti,ab OR bullied:ti,ab OR hostility:ti,ab OR hostile:ti,ab OR aggression:ti,ab OR aggressive:ti,ab OR civility:ti,ab OR civilized:ti,ab OR civilised:ti,ab OR incivility:ti,ab OR uncivil:ti,ab OR disrespect:ti,ab OR disrespectful:ti,ab OR Rude:ti,ab OR Rudeness:ti,ab OR Haze:ti,ab OR Hazing:ti,ab OR belittle:ti,ab OR belittling:ti,ab OR Demean:ti,ab OR Demeaning:ti,ab OR Harass:ti,ab OR harassing:ti,ab OR Anger:ti,ab OR Vulgar:ti,ab OR Vulgarity:ti,ab OR Inappropriate:ti,ab OR Trolling:ti,ab OR Troll:ti,ab OR unprofessional:ti,ab OR professionalism:ti,ab OR misconduct:ti,ab OR misbehavior:ti,ab OR misbehaviors:ti,ab OR misbehave OR 'interpersonal deviance':ti,ab OR disruptive:ti,ab | 425,986 |
| 3 | 'medical education'/exp OR 'paramedical education'/exp OR 'pharmacy school'/exp OR 'medical school'/exp OR 'health student'/exp OR 'continuing education'/exp OR 'faculty practice'/exp OR ((Nursing:ti,ab OR medical:ti,ab OR dental:ti,ab OR pharmacy:ti,ab OR 'public health':ti,ab OR 'allied health':ti,ab OR midwifery:ti,ab) AND (education:ti,ab OR school:ti,ab OR schools:ti,ab OR student:ti,ab OR students:ti,ab OR faculty:ti,ab OR universities:ti,ab OR university:ti,ab)) | 764,820 |
| 5 | #1 AND #2 AND #3 | 998 |
| 6 | #5 NOT (((teens:ti,ab OR teen:ti,ab OR teenager:ti,ab OR adolescent:ti,ab OR adolescents:ti,ab OR adolescence:ti,ab OR child:ti,ab OR kid:ti,ab OR kids:ti,ab OR children:ti,ab OR youth:ti,ab OR 'high school':ti,ab OR infant:ti,ab OR newborn:ti,ab juvenile;ti,ab OR juvelines;ti,ab OR 'juvenile'/exp)) NOT 'adult'/exp) | 926 |
| 7 | #6 AND English:la | 906 |
| 8 | #7 AND [2007-2019]/py | 818 |
| 9 | #8 NOT [medline]/lim | 361 |
| 10 | Additional articles added with update Jan 1, 2020 – March 31, 2020 | 14 |

**Database: ProQuest Dissertations & Theses Global**

| Set # |  | Results |
| --- | --- | --- |
| 1 | SU.EXACT(Internet) OR SU.EXACT(Distance Education) OR SU.EXACT(distance learning) OR SU.EXACT(Virtual learning environments) OR TI (Cyber OR "internet" OR Online OR Web OR "distance " OR Forum OR Forums OR "Discussion Board" OR "Discussion Boards") OR AB (Cyber OR "internet" OR Online OR Web OR "distance" OR Forum OR Forums OR "Discussion Board" OR "Discussion Boards" ) | 675,033 |
| 2 | TI (Cyber-bullying OR cyberbullying OR "cyber aggression" OR “cyber-incivility” OR bullying OR bully OR bullies OR bullied OR hostility OR hostile OR aggression OR aggressive OR civility OR civilized OR civilised OR incivility OR uncivil OR disrespect OR disrespectful OR Rude OR Rudeness OR Haze OR Hazing OR belittle OR belittling OR Demean OR Demeaning OR Harass OR harassing OR Anger OR Vulgar OR Vulgarity OR Inappropriate OR Trolling OR Troll OR unprofessional OR professionalism OR misconduct OR “interpersonal deviance” OR misbehavior OR misbehaviors OR misbehave OR disruptive) OR AB (Cyber-bullying OR cyberbullying OR "cyber aggression" OR “cyber-incivility” OR bullying OR bully OR bullies OR bullied OR hostility OR hostile OR aggression OR aggressive OR civility OR civilized OR civilised OR incivility OR uncivil OR disrespect OR disrespectful OR Rude OR Rudeness OR Haze OR Hazing OR belittle OR belittling OR Demean OR Demeaning OR Harass OR harassing OR Anger OR Vulgar OR Vulgarity OR Inappropriate OR Trolling OR Troll OR unprofessional OR professionalism OR misconduct OR “interpersonal deviance” OR misbehavior OR misbehaviors OR misbehave OR disruptive) OR SU.EXACT(bullying) OR SU.EXACT(aggressiveness) OR SU.EXACT(hazing) OR SU.EXACT(Conflict) OR SU.EXACT(Anger) | 376,245 |
| 3 | SU.EXACT(medical schools) OR SU.EXACT(medical residencies) OR SU.EXACT(dental schools) OR SU.EXACT(nursing schools) OR SU.EXACT(nursing education) OR SU.EXACT(graduate students) OR SU.EXACT(graduate education) OR SU.EXACT(Undergraduate Education) OR SU.EXACT(College faculty) OR SU.EXACT(college professors) OR SU.EXACT(College Students) OR (TI(Nursing OR medical OR "allied health" OR dental OR midwifery OR pharmacy OR "public health") AND TI(education OR school OR schools OR student OR students OR faculty)) OR (AB (Nursing OR medical OR "allied health" OR dental OR midwifery OR pharmacy OR "public health") AND AB(education OR school OR schools OR student OR students OR faculty)) | 36,291 |
| 5 | #1 AND #2 AND #3 | 15,667 |
| 6 | #5 NOT (TI (teens OR teen OR teenager OR adolescent OR adolescence OR adolescents OR child OR kid OR kids OR children OR youth OR "young adult" OR "young adults" OR high school OR infant OR newborn) OR AB (teens OR teen OR teenager OR adolescent OR adolescence OR adolescents OR child OR kid OR kids OR children OR youth OR "young adult" OR "young adults" OR high school OR infant OR newborn)) NOT (SU(adult)) | 659 |
| 7 | #5, Filters: English language; 2000-2019 | 477 |
| 8 | Additional articles added with update Jan 1, 2020 – March 31, 2020 | 4 |

**Education Full Text (H.W. Wilson)**

| Set # |  | Results |
| --- | --- | --- |
| 1 | DE "Internet in higher education" OR DE "Distance Education" OR DE "Nonformal education" OR DE "Alternative education" OR DE "Online courses OR DE "Virtual classrooms" OR DE "Virtual universities & colleges" OR DE "Blended learning" OR DE "Web-based instruction" OR TI (Cyber OR distance OR "internet" OR Online OR Web OR Forum OR Forums OR "Discussion Board" OR "Discussion Boards") OR AB (Cyber OR distance OR "internet" OR Online OR Web OR Forum OR Forums OR "Discussion Board" OR "Discussion Boards") | 12,282 |
| 2 | TI (Cyber-bullying OR cyberbullying OR "cyber aggression" OR “cyber-incivility” OR bullying OR bully OR bullies OR bullied OR hostility OR hostile OR aggression OR aggressive OR civility OR civilized OR civilised OR incivility OR uncivil OR disrespect OR disrespectful OR Rude OR Rudeness OR Haze OR Hazing OR belittle OR belittling OR Demean OR Demeaning OR Harass OR harassing OR Anger OR Vulgar OR Vulgarity OR Inappropriate OR Trolling OR Troll OR unprofessional OR professionalism OR misconduct OR “interpersonal deviance” OR misbehavior OR misbehaviors OR misbehave OR disruptive) OR AB (Cyber-bullying OR cyberbullying OR "cyber aggression" OR “cyber-incivility” OR bullying OR bully OR bullies OR bullied OR hostility OR hostile OR aggression OR aggressive OR civility OR civilized OR civilised OR incivility OR uncivil OR disrespect OR disrespectful OR Rude OR Rudeness OR Haze OR Hazing OR belittle OR belittling OR Demean OR Demeaning OR Harass OR harassing OR Anger OR Vulgar OR Vulgarity OR Inappropriate OR Trolling OR Troll OR unprofessional OR professionalism OR misconduct OR “interpersonal deviance” OR misbehavior OR misbehaviors OR misbehave OR disruptive) OR DE "Bullying" OR DE "Bullying in universities & colleges" OR DE "School bullying" OR DE "Aggression (Psychology)"OR DE "hazing" OR DE "Student attitudes" OR DE "Nursing student attitudes" OR DE "Teacher attitudes" | 33,424 |
| 3 | DE "Graduate medical education" OR DE "Nursing education" OR DE "Associate degree nursing education" OR DE "Baccalaureate nursing education" OR DE "Continuing education of nurses" OR DE "Nursing education (Graduate)" OR DE "Nurse educators" OR DE "Nursing schools" OR DE "Nursing students" OR DE "Nursing teachers" OR DE "Pharmaceutical education" OR DE "Pharmacy colleges" OR DE "Paramedical education" OR DE "Education of physicians' assistants" OR DE "Physical therapy education" OR DE "Health occupations schools" OR DE "Chiropractic schools" OR DE "Dental schools" OR DE "Medical schools" OR DE "Public health schools" OR DE "Health occupations school faculty" OR DE "Health occupations students" OR DE "Continuing education of nurses" OR DE "Medicine -- Study & teaching (Continuing education)" OR DE "College teachers" OR TI ((Nursing OR medical OR "allied health" OR dental OR midwifery OR pharmacy OR "public health") AND (education OR school OR schools OR student OR students OR faculty OR universities OR university)) OR AB ((Nursing OR medical OR "allied health" OR dental OR midwifery OR pharmacy OR "public health") AND (education OR school OR schools OR student OR students OR faculty OR universities OR university)) | 28,454 |
| 4 | #1 AND #2 AND #3 | 43 |
| 5 | #4 NOT ((TI (teens OR teen OR teenager OR adolescent OR adolescence OR adolescents OR child OR kid OR kids OR children OR youth OR “high school” OR infant OR newborn) OR AB (teens OR teen OR teenager OR adolescent OR adolescence OR adolescents OR child OR kid OR kids OR children OR youth OR “high school” OR infant OR newborn) OR DE "Teenagers" OR DE "Youth" OR DE "Adolescence" OR DE "Children" OR DE "High school students")) NOT DE "Adults") | 41 |
| 6 | #5 AND LA English | 41 |
| 7 | Limit publication date to 2000-2019 | 41 |
| 8 | Additional articles added with update Jan 1, 2020 – March 31, 2020 | 3 |

**EthOS eThesis Online Service (**[https://ethos.bl.uk](https://ethos.bl.uk/))

| Set # |  | Results: 2000-2019 | 1/1/2020 –3/31/2020 |
| --- | --- | --- | --- |
| 1 | “online education” | 29 | 33 |
| 2 | online education medical | 53 | 61 |
| 3 | online education health | 113 | 130 |
| 4 | “distance education” | 54 | 59 |
| 5 | distance education medical | 16 | 18 |
| 6 | distance education health | 50 | 55 |
| 7 | internet education medical | 17 | 21 |
| 8 | internet education health | 49 | 59 |
| 9 | incivility | 20 | 23 |
| Total | | 401 | 459 |

**OpenGrey (**<http://www.opengrey.eu>)

| Set # |  | Results: 2000-2019 | 1/1/2020 – 3/31/2020 |
| --- | --- | --- | --- |
| 1 | discipline:(06*) Online education lang:"en" | 6 | 6 |
| 2 | discipline:(06*) distance education lang:"en" | 5 | 5 |
| 3 | discipline:(06*) internet education lang:"en" | 6 | 6 |
| 4 | discipline:(06*) incivility lang:"en" | 0 | 0 |
| Total | | 17 | 17 |

**Appendix II.** Summary of included studies and main findings (n=8)

| **Author(s) (year)/ Country of origin** | **Discipline – Participants, sample size (n)/Domain of cyber environment** | **Participant Characteristics** | **Study Design** | **Aims** | **Description of main results** |
| --- | --- | --- | --- | --- | --- |
| Bork (2014) [28]/USA | Nursing – undergraduate male students (n = 14)/Online classroom | Male students (n=14, 100%) who had taken at least one online nursing course  Participants came from three nursing schools in the midwestern United States of America (U.S.)  Age groups:   - 18 to 24 (n=4) - 25 to 34 (n=6) - 35 to 44 (n=3) - 45 to 54 (n=1) | Qualitative, phenomenological study | Explored faculty behaviors perceived as caring or uncaring by male nursing students interviewed, and the impact of faculty behaviors on students’ motivation and ability to learn and complete the program | Faculty were perceived as caring when they responded quickly to emails (theme 1) and assignment questions (theme 2), and were willing to use diverse methods of communication such as phone calls, text messages, and face-to face meetings to interact with students (theme 3). Male students did not perceive gender bias from faculty (theme 4).  Uncaring behaviors by faculty were described as slow responses to student concerns (theme 1), short responses to emails (theme 2), and vague assignment directions (theme 3). Some students did not perceive any uncaring behaviors by nursing faculty (theme 4).  Caring behaviors by faculty made the learning environment more supportive by motivating students to do better (theme 1), to be more successful (theme 2), and to complete assignments correctly (theme 3).  Uncaring behaviors led students to perceive the learning environment as unsupportive by making them less motivated and more likely to drop out or withdraw from a course (theme 1), and by making it difficult to interact with faculty (theme 3). Some students did not experience uncaring behaviors (theme 2). |
| Cain (2017) [30] /USA | Nursing – undergraduate students (n=246)/Online classroom | Students in an online RN to BSN program in the southwestern U.S.  Female 89.8% (n=221)  Mean age=43.73 (23-80) years old | Quantitative correlational design | Underlying factor structure in uncivil faculty behaviors in the IOLE (Incivility in Online Learning Environment)  Determining relationships between age, gender, and uncivil behaviors in online classes | A two-factor structure labeled unprofessional faculty behavior (accounting for 79.3% of variance) and classroom management (accounting for 5.6% of variance) as underlying uncivil faculty behaviors in the IOLE.  No correlation was found between gender and either of the two new IOLE factor scores: unprofessional faculty behavior (r_pb_=-.023, p=.72), and classroom management (r_pb_=-.037, p=.56).  No correlation was found between age and either of the two new IOLE factor scores: unprofessional faculty behavior (r_pb_=.05, p=.45), and classroom management (r_pb_=.06, p=.29. |
| De Gagne et al. (2018) [9]/USA | Various health professions graduate students (n=25)/ Email, social networking sites, online discussion forums | Students in nursing 56% (n=14), Doctor of Medicine 24% (n=6), Physical Assistant 12% (n=3), and Doctor of Physical Therapy 8% (n=2) programs at a private university in the southeastern region of the U.S.  Female 84% (n=21)  Mean age=34.3±8.9 (25-56) years | Qualitative descriptive design | Explored perceptions on the concept of cybercivility and cyberincivility, experiences with cyberincivility, and perspectives on interprofessional cybercivility learning | Perceptions of concepts related to cybercivility and cyberincivility: Behavior that is disrespectful, unprofessional, hurtful, unkind, bullying, or rude. Code of ethics should be applied.  Experiences with cyberincivility (multiple platforms): Examples included negative comments, violations of privacy, misuse of reviews, rude or terse emails (such as passive aggressive tones), or overusing the ‘reply all’ feature. Triggers for incivility were related to stress, lack of cues, and information overload. Consequences of incivility were communication breakdown leading to negative personal and care consequences.  Strategies for fostering cybercivility: Regular training, rules and regulations, maintain professional distance, role models, and rereading before sending/posting. |
| Hart & Morgan (2010) [32]/USA | Nursing – undergraduate students (n=374)/Online classroom | Traditional classroom students (n=44)  Female 84.1% (n=37)  Age = 47.7% (n=21) 40 years old or younger  The traditional cohort of students resided in the same region of the southeastern U.S., and the online cohort consisted of students located throughout the U.S. and internationally.  Online classroom students (n=330): female: 93.3% (n=305); age: 30.4% (n=100) 40 years or younger  Online classroom students were significantly older than traditional classroom students (x^2^=5.32, df=1, p=.02) | Quantitative comparative descriptive design | Scores on a modified Donald McCabe Academic Integrity Survey:   - Frequency of cheating - Rank the effectiveness of policies - Rank the seriousness of cheating actions - Experience of witnessing others cheating | Both groups reported very low levels of cheating experiences and very high standards of academic integrity.  No significant difference between groups on frequency of plagiarism, inappropriate sharing of work in group assignments, or cheating on tests.  Online students had a better understanding of campus policies (t(46)=3.37, p<.01) and of student support for policies (t(44)=2.81, p=.01).  No difference in how groups ranked severity of penalties for cheating (t(44)=.961, p=.34).  Online students were more likely to identify these behaviors as serious: Working with other students on individual assignments (χ^2^=19.00, df=3, p<.01), working with other students via email or instant messaging (χ^2^=8.250, df=3, p=.04), obtaining answers to test questions from others (χ^2^=21.67, df=3, p<.01), copying from another student during testing (χ^2^=12.8, df=3, p<.01), copying another student’s homework (χ^2^=11.7, df=3, p<.01), and receiving unpermitted help (χ^2^=10.6, df=3, p=.01).  Online students learned academic integrity more from orientation (χ^2^=18.20, df=2, p<.01), program website (χ^2^=47.70, df=2, p<.01), and student handbook (χ^2^=12.50, df=2, p<.01). |
| Morgan & Hart (2013) [33]/ USA | Nursing – undergraduate students (n=346)/Online classroom | Online RN to BSN nursing students: control group (n=169) and intervention group (n=177)  Female 92.7%  Age: 97% 24 years or older | Quasi-experimental design | Scores on a modified Donald McCabe Academic Integrity Survey:   - Frequency of cheating - Rank the effectiveness of policies - Rank the seriousness of cheating actions - Experience of witnessing others cheating | No significant difference was found on self-reported cheating between the two groups (z=-9.41, p>.05).  No significant difference was found on the seriousness of cheating between the two groups (z=-.883, p>.05).  Intervention group reported more faculty support (z=-2.87, p <.05) and student support (z=-2.37, p<.05) than the control group.  Intervention group perceived higher levels of understanding for academic integrity policies by faculty (z=-2.04, p<.05) and perceived academic integrity polices to be more effective (z=-2.48, p<.05) than the control group. |
| Rieck & Crouch (2007) [29] /USA | Nursing – undergraduate students (n=96)/Online classroom | Traditional (46.9%, n=45), RN to BSN (36.9%, n=35), Fast track (10.4%, n=10), Reservation (4.2%, n=4), No response (2.1%, n=2)  Participants were students enrolled in online nursing courses in a university in the southwestern region of the U.S. | Qualitative exploratory-descriptive design | Sense of connectiveness with peers and with the instructor  Perceptions about uncivil communication  Instructor’s techniques for managing rude communication | Connectiveness with peers:   - Fixed responses: online discussion (52%, n=50), face to face instruction (46%, n=44), meeting at beginning (41%, n=39) - Open-ended: face to face instruction (30%, n=17), social strategies (17%, n=9.5), discussion and chats (16.1%, n=9)   Student and faculty connectiveness:   - Fixed responses: timely response (71%, n=69), meeting at beginning of semester (62%, n=60), use of phone (32%, n=31) - Open-ended: face to face instruction (30%, n=17), timely feedback (29.5%, n=16.5), social strategies (17%, n=9.5)   Uncivil communication:   - Experienced rude communication (61%) - Rude experience with students (35%) and with faculty (60%) - 63% students felt faculty did not handle uncivil behavior well. - 49% students felt uncivil behavior should be handled in a private manner. |
| Skrabal (2017) [34]/ USA | Nursing – undergraduate students (n=12)/Social media (Facebook, Twitter, Instagram, Snapchat) | Age groups:   - 19 to 22 (n=5) - 23 to 26 (n=4) - 27 to 30 (n=2) - 31 to 35 (n=1)   Participants came from two nursing schools in the midwestern U.S. | Qualitative grounded theory | The process of maintaining e-professionalism in social media among prelicensure baccalaureate nursing students | E-professionalism (consequences): recognizing e-professional behaviors and processes when creating professional postings  Maintain privacy boundaries (strategy): avoiding posting information, using different social media accounts, and providing social media education and resources  Factors influencing e-professionalism: age, formal education through nursing school, ethical reasoning, managing emotions, searchability - the ability to find anyone or anything in social media, and daily use |
| Smith (2010) [31]/ USA | Nursing – undergraduate students (n=150)/Online classroom  Tool development done with interviewing 7 online RN-BSN students  A pilot study with18 online RN-BSN students  Reliability and validity testing with 125 online RN-BSN students | Online RN-BSN students across the U.S.  The majority of participants were female (89.9%), White (79.5%), 18-40 years old (55%), and working full time (76.5%). | Quantitative descriptive-exploratory study | A relationship between academic and professional dishonesty behaviors in online nursing students | Academic dishonesty behaviors were not impacted by age, ethnicity, gender, or work.  Students who worked full time were more likely to have committed professional dishonesty within the last 365 days (χ^2^ =10.6615, df=1, p=.0011).  Students of schools with an honor code were less likely to commit academic dishonesty (χ^2^ =5.9263, df=1, p=.0149).  Students with less experience of working as a RN were more likely to engage in acts of academic dishonesty (χ^2^ =7.6063, df=1, p=.0223).  68% on online RN-BSN students had engaged in behaviors of academic dishonesty, most commonly by not contributing to group work (85.5%). Nearly 90% had engaged in professional dishonesty, with incivility being the most common (76.8%).  A positive relationship existed between academic and professional dishonesty (r=0.43855, p<.0001).  Acts of plagiarism were correlated with theft (r=0.27115, p=.013) and with misuse of information (r=0.24030, p=.0045).  Students who felt academic dishonesty behaviors were ethical had increased likelihood of engaging in academic dishonesty (r=0.270, p=.001) and professional dishonesty (r=.333, p=.000).  Students who intended to engage in dishonest behavior were more likely to engage in academic dishonesty (r=0.288, p=.00) and professional dishonesty (r=0.273, p=.001). |
